# Diallel Cross Application and Histomolecular Characterization: An Attempt to Develop Reference Stock of *Labeo ariza*

**DOI:** 10.3390/biology11050691

**Published:** 2022-04-30

**Authors:** A. K. Shakur Ahammad, Neaz A. Hasan, Abul Bashar, Mohammad Mahfujul Haque, Muyassar H. Abualreesh, Md. Mehefuzul Islam, Biraj Kumar Datta, Md. Fazla Rabbi, Mohd Golam Quader Khan, Md. Samsul Alam

**Affiliations:** 1 Department of Fisheries Biology and Genetics, Bangladesh Agricultural University, Mymensingh 2202, Bangladesh; mehefuz.40622@bau.edu.bd (M.M.I.); biraj.40527@bau.edu.bd (B.K.D.); rabbi.39314@bau.edu.bd (M.F.R.); khanmgq@bau.edu.bd (M.G.Q.K.); samsul.alam@bau.edu.bd (M.S.A.); 2 Department of Aquaculture, Bangladesh Agricultural University, Mymensingh 2200, Bangladesh; neaz41119@bau.edu.bd (N.A.H.); bashar43791@bau.edu.bd (A.B.); mmhaque.aq@bau.edu.bd (M.M.H.); 3 Department of Marine Biology, Faculty of Marine Sciences, King Abdulaziz University, Jeddah 22254, Saudi Arabia; mabulreesh1@kau.edu.sa

**Keywords:** diallel cross, *Labeo ariza*, growth performance, muscle histology, genetic variation, microsatellite markers

## Abstract

**Simple Summary:**

Diallel crosses are breeding schemes used to estimate combining ability and the effects of reciprocal crosses among strains or varieties. Using this diallel cross scheme, we produced a crossbred population of Ariza labeo (*Labeo ariza*), a newly potential aquaculture species in Bangladesh. The improved performances of this crossbreed were observed phenotypically (growth performances) and genetically (variation at selected microsatellite loci) and manifested through myofiber histology. Captive breeding of this species in carp hatcheries of Bangladesh could be initiated and facilitated through maintaining the gene pool of the *L. ariza* of Kangsha and the Atrai River. Conservation could also be considered for advancing the better performer crossbred (G4K♀A♂) as the reference diallel crossbred. The simple yet efficient breeding technique and the manifestation of growth performances using histological and phenotypical observations will encourage the hatchery operators of Bangladesh and elsewhere to initiate the induced breeding of *L ariza* in large scale aquaculture.

**Abstract:**

The objective of the present study was to evaluate the growth performance and genetic variation in diallel crosses of Ariza labeo (*Labeo ariza*) originating from three geographically separated rivers (Atrai, Jamuna and Kangsha) in Bangladesh. Intra (G1K♀K♂, G2J♀J♂, and G3A♀A♂) and inter (G4K♀A♂, G5K♀J♂, G6A♀K♂, G7A♀J♂, G8J♀K♂, and G9J♀A♂) stocks were produced following diallel cross (sex ratio—1:1 and *n* = 48; 16 from each river). Reproductive and growth performance, muscle cellularity and genetic variation following genotyping of eight microsatellite markers (*Lr1*, *Lr2*, *Lr3*, *Lr22*, *Lr24*, *Lr27*, *Lr28* and *Lr29*) and analysis of all crossbreeds was performed. The fertilization (95% ± 2.11%), hatching (88% ± 1.03%), and survival rates (82% ± 1.88%) of G4K♀A♂ were higher compared to other groups. With respect to length and weight gains (2.67 ± 0.4 cm and 3.39 ± 0.2 g), SGR (3.23% ± 0.20%), and heterosis (8.87% and 24.74%) G4K♀A♂ was the superior group. A higher number of hyperplastic muscle fibers, mean number of alleles (2.75) and mean observed heterozygosity (0.417) from G4K♀A♂ could be interpreted to mean that G4K♀A♂ comprise better performance efficiency compared to others and are considered for continuing the *L. ariza* stock improvement program.

## 1. Introduction

Ariza labeo (*Labeo ariza*) (Hamilton, 1807) is considered one of the most commercially important indigenous species (belongs to minor carp group) in the Asian subcontinent including Bangladesh, and it inhabits small rivers, floodplains, and creeks throughout the Indo-Pacific region [1,2]. *L. ariza* is an omnivore and column feeder that grows to a maximum size of 50 cm in length [3] and attains sexual maturity at over 1 year of age [4]. This fish has generated much interest in Bangladeshi aquaculture owing to its high nutritional value, which is relatively higher than that of the major Indian carps (Ruhu: *Labeo rohita*; Catla: *Catla catla*; Black Rohu: *Labeo calbasu*; Mrigal: *Cirrhinus cirrhosus*), and high market demand [5]. *L. ariza* has been listed as a vulnerable fish species in Bangladesh [6]. The development of induced breeding techniques has brought considerable attention to the production of offspring for large-scale aquafarming and to the conservation of this species. These same techniques were adopted for offspring production of other cyprinids including *Dawkinsia rohani* (Rohan’s barb) [7], *Osteobrama belangeri* (Pengba) [8] and *Labeo gonius* (Minor carp) [9]. However, the offspring produced through induced breeding in the hatcheries have often been reported to have growth and survival issues owing to a variety of factors, including the selection of breeding populations and breeding techniques, where scientific interventions are deemed necessary [10,11,12]. These limiting issues could be optimized through proper selection of spawning agents [13], the comprehensive investigation of several areas of early larval biology [14] and the improvement of existing breeding techniques [15].

Diallel crossing is a breeding scheme that has been extensively applied to estimate the combining ability as well as the effect of reciprocal crosses in plants and animals [16,17,18]. This mating scheme has been used to explore the genetic underpinnings of quantitative traits by geneticists and breeders [19]. The improvement of economically important traits (such as growth), while also gaining sufficient genetic variation, is one of the prime goals in contemporary aquaculture. In culturable species such as *L. ariza*, where the success of breeding schemes is countered by the complexities of negative selection (e.g., spawning late-matured fish) and inbreeding, a diallel mating design could help improve the genetic and phenotypic gains and may offer additional management tools to develop the base population of a breeding program [20,21]. Healthy and rapidly growing fry and fingerlings are crucial biological components for the competitive expansion of the aquaculture industry that targets enhanced production [22,23,24]. Muscle growth serves as an important indicator to identify and select superior fish fry and/or fingerlings for stocking in grow-out farms. The myofiber growth of fish evidently affects growth and size through the fiber surface area-to-length ratios which control the nutrient assimilation rates of fish [25,26].

Muscle growth in fish develops through the generation of new muscle fibers (hyperplasia—a condition that augments cell numbers of a particular organ and/or tissue) and/or the expansion of existing muscle fibers (hypertrophy—An increase in the size of cells (or tissues) in response to various stimuli) [26,27,28]. Rapid growth to large size in fish results from a sustained recruitment of new fibers into their axial series of myomeres [29]. The cessation of recruitment at a small fish size leads to slow growth and a small final size of the fish. Any genetic variation gained by the diallel mating system in the founder population may be evident through enhanced myofiber growth in the progeny [30]. The relative size and shape of myofibers during the earlier stage of development revealed through muscle histology could be indicators of the success (or failure) of a breeding or selection program. One of the most common uses of genetic markers in fisheries biology is to determine the nature and level of genetic variation in terms of polymorphisms and heterozygosity. Microsatellite DNA markers are powerful tools and are widely and efficiently used to assess genetic variation in fish populations [31,32,33,34]. Microsatellite analysis has provided essential information for the formulation of meaningful conservation strategies for fisheries and aquaculture management [35].

Considering the threatened status of *L. ariza* in the wild and the long history of poor outcomes of the breeding schemes in the carp hatcheries of Bangladesh [36], we implemented a diallel crossing scheme in selected stocks of *L. ariza* and comprehensively studied the performance of the intraspecific crossbred populations based on physical growth and myofiber histology. We also assessed the molecular genetic variation in crossbreeds using microsatellite DNA markers. Therefore, the aim of the present study was to evaluate the growth performance and genetic variation in diallel crosses of the Ariza labeo (*L. ariza*) originating from three geographically separated rivers in Bangladesh. We also aimed to conserve the best group as a founder stock that will facilitate future research aiming to promote sustainable genetic and phenotypic gains in *L. ariza* populations.

## 2. Materials and Methods

### 2.1. Ethical Statement

This study included sampling from the wild, breeding in captivity, and fin clipping of the animals without euthanizing them, among other activities. Animal scientific procedures were rigorously followed during these activities with prior approval by the Animal Welfare and Ethics Committee of the Bangladesh Agricultural University (BAU) (ref. no. BAURES/ESRC/FISH-30/22).

### 2.2. Collection and Rearing of Wild L. ariza

**Figure 1:**
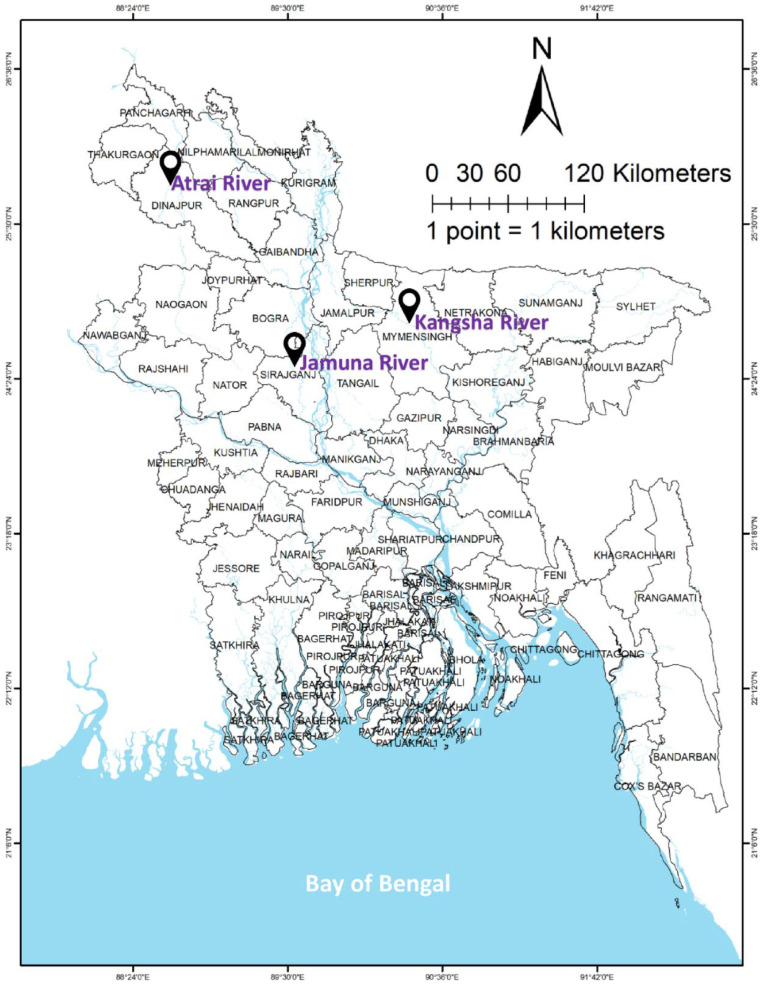
Geographical position of three rivers belonging the Ariza labeo (*L. ariza*) population from where the sample fry was collected.

Advanced fry of *L. ariza* (*n* ≥ 900) were collected from each of the three river populations of Bangladesh, i.e., the Atrai (Dinajpur District), Kangsha (Mymensingh District), and Jamuna Rivers (Sirajganj District) (Figure 1). The average length (cm) and weight (g) of the fish were 5.89 ± 0.85 cm and 7.08 ± 0.94 g, 5.80 ± 0.73 cm and 7.06 ± 0.91 g, and 5.85 ± 0.92 cm and 7.01 ± 0.86 g, respectively. They were stocked in nine ponds (18 × 14 × 1.3 m each) equipped with inlet-outlet, water exchange, and aeration systems. The fish were subjected to three treatments with three replications each. All experiments were performed in the Field Laboratory Complex of the Faculty of Fisheries, BAU. The fish were reared for six months and were given feed containing 35% protein (Mega Feed Ltd., Dhaka, Bangladesh) twice daily at approximately 5% of their body weight. Major water quality parameters were recorded weekly; temperature was measured using a Celsius thermometer (SMART SENSOR* AR867, Dongguan, China); dissolved oxygen was measured using a dissolved oxygen meter (DO-5509, Lutron, Taipei, Taiwan), and ammonia was measured using ammonia test strips (Indigo instruments, Waterloo, ON, Canada); nitrite, alkalinity, hardness, chlorine, and pH were measured using water test strips (Thomas Scientific, Swedesboro, NJ, USA).

### 2.3. Selection of L. ariza for Grow-Out Culture

One hundred and eighty *L. ariza* (late juvenile stage with 1:1 sex ratio) from each stock (mean length and weight, 10.23 ± 0.25 cm and 53.52 ± 0.43 g, respectively) were reared for six months in nine separate grow-out ponds (18 × 14 × 1.3 each). A vitamin E supplement was incorporated into the protein-rich feed (35%) during the rearing period and fed at 5% of the body weight. Consequently, after 12 months of culturing (including six months of nursery rearing and six months of grow-out culture), the final length, weight, and survival rates of the fish were determined.

### 2.4. Determination of the Gonadosomatic Index (GSI)

The GSI, a metric representing the relative weight of the gonad to the fish body weight, was determined over a period of 12 months for mature fish. Year-round (between October 2017–September 2018) GSI data was collected following the sacrifice of three males and three females [37] in every month using the following formula:

(1) $$GSI=\frac{Gonad\;weight\;of\;fish}{Live\;body\;weight\;of\;fish}\times 100$$

### 2.5. Diallel Crossing among Individuals of the Three River Populations

A total 48 broods (16 from each stock source from three riverine system following a sex ratio of 1:1) were selected for a 3 × 3 diallel crossing, within and among stocks of the Kangsha, Jamuna, and Atrai Rivers. The pituitary extract of carp was used for effective induced breeding from selected brood samples. Female broods (having an average length 16.34 ± 0.44 cm and weight 90.13 ± 0.23 g) were treated with two doses (4 mg·kg^−1^ bw and 8 mg·kg^−1^ bw) at 6-h intervals, while the males (their average length and weight were 14.51 ± 0.29 cm and 89.24 ± 0.11 g, respectively) were treated with a single dose (4 mg·kg^−1^ bw) at times when the females received their second dose.

**Figure 2:**
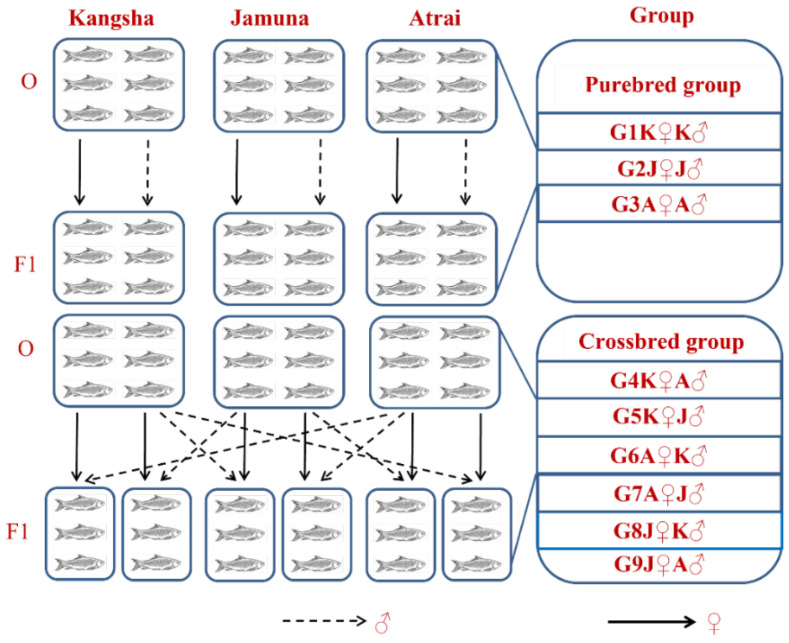
Inter- and intra-species ordination of different sample populations of Ariza labeo (*L. ariza*) to propagate and procreate F1 generation. The single words picked for naming the purebred and crossbred groups bear the full forms as G → Group, K → Kangsha River stock, J → Jamuna River stock and A → Atrai River stock.

We designated the intraspecies inter- (and intra) stock crosses to produce the F1 generation and the groups were acronymed as G1K♀K♂, G2J♀J♂, G3A♀A♂, G4K♀A♂, G5K♀J♂, G6A♀K♂, G7A♀J♂, G8J♀K♂, and G9J♀A♂ (For details see Supplementary Materials and Figure 2). The fertilized eggs were transferred into glass aquaria (10 L each) with continuous water flow. An optimum temperature of 28.0 °C was maintained using thermostat heaters (FZ-628, Weifang Yipin Pet Products Co. Ltd., Shandong, China) for 21 days. The reproductive performance (e.g., hatching and survival rates) of each group was determined at this stage.

### 2.6. Growing out Crossbred Groups of L. ariza and Determination of Heterosis

Twenty-one-day-old fry were released in nine different earthen ponds (9 × 7 × 1.3 m each) with the same facilities as those described in Section 2.1, maintaining a stocking density of 800 in each pond for a period of 60 days with ad libitum feeding. Gains in length, weight, and specific growth rate (SGR) were determined after this period. The growth performance of 20 fish from each group was determined, and heterosis was calculated according to the method proposed by Tave [38].

### 2.7. Muscle Histology of Different L. ariza Groups

The muscle growth patterns of early juveniles (82 days post fertilization) of both the intra- and inter-stock crossbreds were analyzed using trunk muscle histology counterstained with hematoxylin [27,39]. Briefly, the ethanol-preserved trunk muscle was subjected to different steps (dehydration, infiltration, and cleaning) of tissue processing in an automatic tissue processor (TP 1020, Leica, Wetzlar, Germany) using a series of alcohol doses of increasing concentrations, two changes of xylene, and finally molten wax. We cut paraffin-embedded blocks (4–5 µm in size); these were kept in a water bath and then placed on a glass slide, which was kept in a slide drier (ASD220, Amos Scientific Pty. Ltd., Melbourne, Australia) overnight. The sections were then stained routinely with hematoxylin [40] and Canada balsam was used for mounting purposes. A compound microscope (Olympus CX41, Olympus, Tokyo, Japan) was used for capturing effective photographs as well as observing, counting, and measuring muscle fibers. Sigma Scan Pro (V. 5.0) software (Systat Software Inc., San Jose, CA, USA) was attached with the microscope for measuring muscle fiber size. Hyperplastic and hypertrophic growth of muscle fibers were measured following the established criteria of the 20 mm diameter method described by Rowlerson and Veggetti [41]. As the measured cross-section areas of muscle fiber are supposed to be circular (πr^2^), these measurements were further converted to the values of equivalent diameter, in accordance with Weatherley and Gill [42]. The growth of muscle fiber was either defined as hyperplasic or hypertrophic when the measurement embeds within the following range:

(2) $$\mathrm{Hyperplasia}<314{\;\mathrm{mm}}^{2}<\;\mathrm{Hypertrophic}$$

Measured muscle fibers were categorized into small (<20 µm), medium (21–30 µm), and large (>30 µm), in conformity with Johnston [27], and for analysis thirty random photographs (best) from each designated group was assessed.

### 2.8. Genetic Characterization of Different L. ariza Groups

Eight microsatellite markers (*Lr1*, *Lr2*, *Lr3*, *Lr22*, *Lr24*, *Lr27*, *Lr28*, and *Lr29*) (Table 1) developed for *L. rohita* [43] were used in this study to determine the population’s genetic variation within and among nine different groups of *L. ariza* originating from diallel crossing. Genomic DNA was extracted from the fin samples (*n* = 270; 30 samples/group) using the GeneJET Genomic DNA Purification Kit (K0721, Thermo Fisher Scientific, Altrincham, UK). The microsatellite loci were amplified using PCR in a 10-μL reaction volume following the standard PCR protocol [44]. For fragment separation, the amplified PCR products were electrophoresed using polyacrylamide gel for approximately 2.5 h at 100 V. The gel was stained with ethidium bromide at a concentration of 0.5 µg/mL (stock solution: 10 mg/mL) and exposed to UV light in the gel documentation system; photographs were taken, and the alleles were scored.

### 2.9. Statistical Analysis

Significant difference of growth indices and muscle fibers mean’ number of different F1 generation groups was calculated by one-way analysis of variance. A multiple range test of Duncan’s at a 5% significance level was performed to specify the difference between groups. These growth and muscle fiber data were analyzed using SPSS (Version 23.0, IBM, Armonk, NY, USA). Alpha Ease FC 4.0 was used to estimate markers and allelic length. The GenAlEx program version 6.51b2 proposed by Peakall and Smouse [45] was used to estimate the number of alleles (N_a_), the effective number of alleles (N_e_), the frequency of alleles, observed heterozygosity (H_o_), expected heterozygosity (H_e_), fixation index (F = 1−Ho/He), Nei’s genetic distance, and gene flow (N_m_).

## 3. Results

### 3.1. Growth Performance of Parental Stock of L. ariza during the Domestication Period

The observed growth performance and survival rates of *L. ariza* originating from three riverine sources after a domestication period of 12 months are shown in Table 2. The final length and weight of the Atrai River stock were significantly higher (*p* < 0.05; 16.78 ± 0.68 cm and 95.92 ± 2.24 g, respectively) than those of the Kangsha (14.36 ± 1.05 cm and 90.36 ± 1.88 g, respectively) and the Jamuna (13.30 ± 3.05 cm and 89.65 ± 2.46 g, respectively) river stocks. The survival rate of Kangsha fish was significantly higher (91% ± 0.3%) than those of the Atrai (86% ± 0.12%) and Jamuna (84% ± 0.15%) fish.

### 3.2. The GSI of L. ariza during the Domestication Period

**Figure 3:**
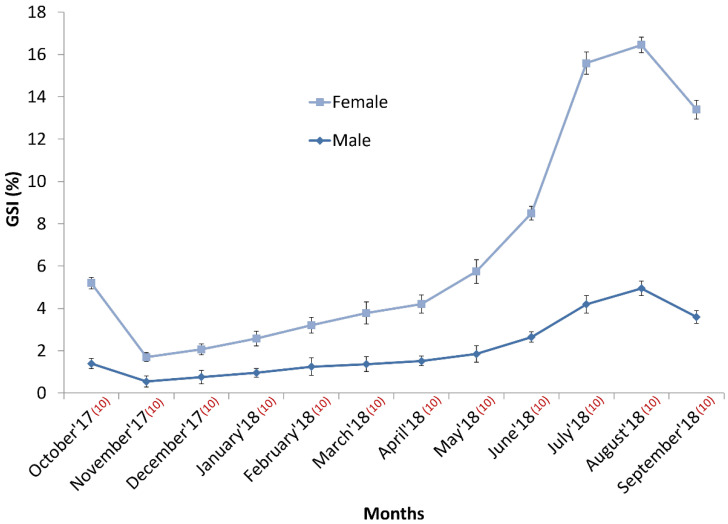
Year-round GIS variation between males and females during in-house domestication of Ariza labeo (*L. ariza*).

Naturally, the GSI values of females becomes more compared to males for wight variation between the ovary and testis. In this experiment, the GSI value of males varies between 0.55 ± 0.26 and 4.95 ± 0.35. As opposed to females, the lower and upper bounds of GSI was 1.15 ± 0.21 and 11.5 ± 0.37, respectively. Coincidentally, August was the peak GSI-recorded month for both sexes. In contrast, the GSI indices of both sexes were the lowest in November. The GSI of males plateaued in April, with a steady increase until May; it then increased gradually for the next three months, peaked in August, and then declined moderately (Figure 3). A similar pattern was evident in females; their GSI increased sharply from April to July and then increased slowly until it peaked the following month.

### 3.3. Reproductive Performance of L. ariza during the Diallel Crossing of the Three Populations

The fertilization (95% ± 2.11%), hatching (88% ± 1.03%), and survival rates (82% ± 1.88%) of G4K♀A♂ were higher (*p* < 0.05) than those of the other groups during seed production. Comparatively lower fertilization and hatching rates were found in G6A♀K♂ and G2J♀J♂ (90% ± 1.99% and 81% ± 4.01%, respectively), with G2J♀J♂ exhibiting the lowest survival rate among all groups (Table 3).

### 3.4. Growth Performance and Heterosis of Crossbreds L. ariza

The length and weight gains were the highest in G4K♀A♂ (2.67 ± 0.4 cm and 3.39 ± 0.2 g, respectively) after 60 days of rearing (*n* = 20 in each group) (Table 4). The lowest length gain (2.40 ± 0.3 cm) and weight gain (3.04 ± 0.2 g) were recorded in G3A♀A♂and G2J♀J♂, respectively. The SGR was significantly higher (*p* < 0.05) in G4K♀A♂ (3.23% ± 0.20%) and lower in G2J♀J♂ (2.98% ± 0.20%). Heterosis, for both length and weight, was higher in G4K♀A♂ (8.87% and 24.74%, respectively) than in the other crossbred groups (Table 5).

### 3.5. Muscle Morphometry of Different Crossbreeds of L. ariza

**Figure 4:**
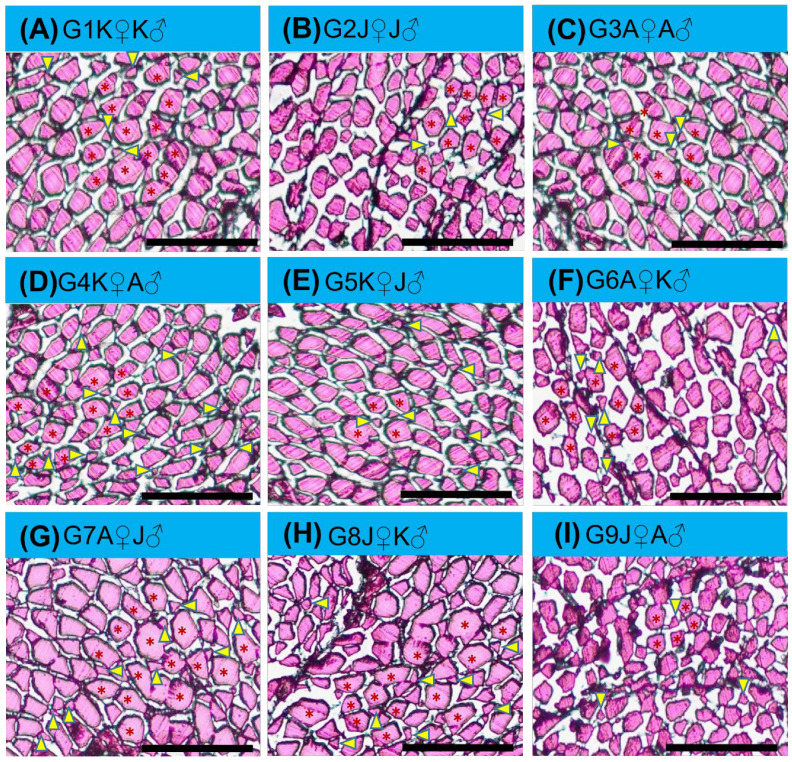
Muscle morphometrical variation of different crossbred groups of Ariza labeo (*L. ariza*) F1 generation. [(**A**), (**B**), (**C**), (**D**), (**E**), (**F**), (**G**), (**H**) and (**I**) represent G1K♀K♂, G2J♀J♂, G3A♀A♂, G4K♀A♂, G5K♀J♂, G6A♀K♂, G7A♀J♂, G8J♀K♂, and G9J♀A♂ groups, respectively. The single words picked to name the purebred and crossbred groups bear the full forms as G → Group, K → Kangsha River stock, J → Jamuna River stock and A → Atrai River stock. Recruited myofibers (hyperplastic) are smaller (yellow arrowheads indicated) situated between the medium and large diameter muscle fiber (red asterisk mark indicated)]. Scale bar = 50 µm.

**Figure 5:**
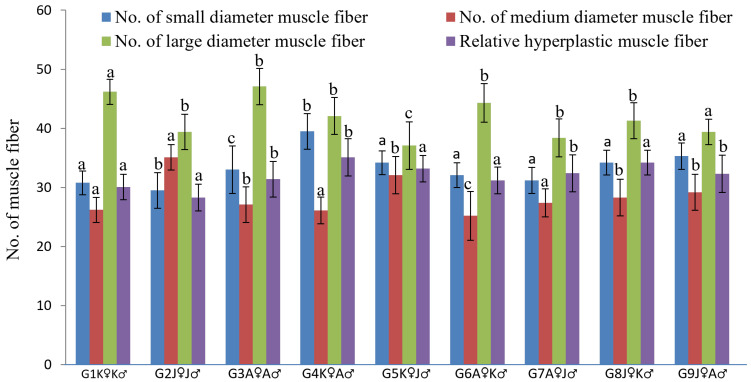
Muscle fiber morphometrical variation of different crossbred groups of Ariza labeo (*L. ariza*) F1 generation (different letters above the error bar indicate the significant difference between the crossbred groups for each parameter). The single words picked to naming the purebred and crossbred groups bear the full forms as G → Group, K → Kangsha River stock, J → Jamuna River stock and A → Atrai River stock.

A mosaic pattern distribution of fibers (with fibers of various diameters) was observed in transverse sectional trunk skeletal muscle histology (Figure 4). Small, medium, and large—for all diameter classes of fiber, each group was identical, and a significant difference was found between all groups. G4K♀A♂ was the highest ranked group where the mean number of small-diameter fibers derived from hyperplastic action was recorded more than those in the other groups (Figure 5).

### 3.6. Genetic Characterization of Different Crossbreds of L. ariza

The microsatellite profiles of loci *Lr3*, *Lr24*, and *Lr27* were identified. Loci *Lr24* and *Lr27* were polymorphic, while loci *Lr1*, *Lr2*, *Lr22*, *Lr28*, and *Lr29* were monomorphic in all groups; *Lr3* was polymorphic in five groups. The average N_a_ (2.75) and average H_o_ (0.417) were the highest in G4K♀A♂. The lowest N_a_ (2.000) and H_o_ (0.167) were observed in G6A♀K♂ and G1K♀K♂, respectively. Negative average fixation indices over all loci were observed in G4K♀A♂ (−0.116), G6A♀K♂ (−0.452), and G7A♀J♂ (−0.144) (Table 6). The highest N_m_ (7.277) was observed between G3A♀A♂ (A♀ × A♂) and G6A♀K♂ (A♀ × K♂), while the lowest N_m_ (0.838) was observed between G1K♀K♂ and G3A♀A♂ (Table 7). The highest and lowest Nei’s genetic distances (0.335 and 0.050, respectively) were observed between G1K♀K♂ and G3A♀A♂ and between G3A♀A♂ and G6A♀K♂, respectively (Table 7).

## 4. Discussion

Sufficient genetic variation in the base population of a selective breeding program is a prerequisite for its success. In the present study, we performed a complete diallel cross to increase the genetic variation and improve the growth performance of *L. ariza*, a newly introduced but popular aquaculture species in Bangladesh. This present study aimed to observe and evaluate the outcomes of a 3 × 3 diallel crossing of riverine populations of *L. ariza* to assess the performance of the progeny in terms of i) the phenotypic gain (or loss) of economically important traits (i.e., growth), ii) muscle fiber morphometry, and iii) genetic variation observed among the crossbred groups. Moreover, following the genetic principles of fisheries management, the superior group attained from this study (if any) aimed to be conserved as a founder population and also aimed to retain or increase the variability of this population.

### 4.1. Growth Response and GSI Performance of Parental Stocks

In this study, the advanced fry of *L. ariza* from three different rivers were collected and reared in on-farm ponds. After 12 months of on-farm rearing, in terms of different growth indices, populations from different riverine systems exhibited the best performance. For length gain and weight gain, the population from the Atrai River was superior, with the population from the Kangsha River having the highest survival rate. The growth trial of the present study was conducted in nine uniform earthen ponds located side by side under similar environmental conditions. All of the major water quality parameters observed during the experiment were within suitable ranges for aquaculture. Therefore, the growth performance variation was likely due to heterozygosity. Bangladesh is a small country with little climatic and environmental variation in its different regions. Samples were collected from the north (Atrai River), central (Jamuna River), and eastern (Kangsha River) regions. Over hundreds of thousands of years, an independent genetic structure evolved in these rivers. Therefore, we conclude that the better performance shown by the Atrai River stock was because of genetic effects. We also found better growth performance in G4K♀A♂. Corroborating our results, higher length and weight gains in *L. ariza* from the Atrai River were also reported by Asaduzzaman [1]. Therefore, this higher growth and survival of Atrai River stock could possibly be attributed to the maintenance of higher levels of heterozygosity in this stock than in the Jamuna and Kangha River stocks.

GSI is helpful in identifying a potential period or season/s of spawning and to ascertain the nature of the spawners (asynchronous or synchronous) that could facilitate the planning and conducting of induced breeding. GSI has also recently been considered as a reference point against any changes in sexual development and/or reproductive success, both in the wild and in captivity [46]. In this study, both sexes had almost identical patterns of increasing and decreasing trends of GSI, which peaked in August; therefore, *L. ariza* can be considered a synchronous spawner, unlike other carps (for example, Chinese carps, *Hypophthalmichthys molitrix* [47] and *Hypophthalmichthys nobilis* [48]), but like major Indian carps (e.g., *Labeo rohita*, *Catla catla*, *Cirrhinus cirrhosus*, and *Labeo calbasu*) and some minor carps and minnows (e.g., *Labeo bata*, *Labeo gonius*, and *Cirrhinus reba*). The results of the present study suggest that *L. ariza* has only one breeding season (between July and September), and it is short. It reaches its peak in August (Figure 3), in contrast to its closest species *Cirrhinus reba*, whose breeding season lasts from June to October [49,50] with a peak in July in Bangladesh waterbodies [51].

### 4.2. Heterosis in the Reproductive and Growth Performances of Crossbred Groups

The higher fertilization, hatching, and survival rates in F1 progeny from G4K♀A♂ might be attributable to the combination of better alleles and the dominant genetic variance. However, the dominant genetic variance is not predictable. In the case of *C. reba* (a species taxonomically close to *L. ariza*) seed production, the application of Ovaprim as an inducing agent was conducive to the fertilization rate (86.4%) and hatching rate (91.6%) at a satisfactory level [52]. The inter-stock crossbreeds in the present study had higher growth performance (in terms of SGR, length, and weight gain) than the intra-stock ones. This may be due to the positive heterosis effect of the heterozygous genotypes created in the crossbred groups owing to a combination of different alleles of the crossing parental populations. Better growth rates were evident in an Egyptian strain of Nile tilapia when a selective breeding scheme using diallel mating design was performed in geographically distant parental populations [53]. An increase in the body weight of the genetically improved farmed tilapia strain was evident in five generations of selection [21]. One of the crossbreeds, G4K♀A♂, showed a higher percentage of positive heterosis (8.87% and 24.74% in terms of length and weight, respectively), resulting in hybrid vigor in the crossbred groups. Shah [34] et al. obtained a similarly high value of heterosis (+21%) when they performed crossbreeding between two stocks of rohu (*L. rohita*) (Hatchery♂ × Padma♀). Positive heterosis in growth was also reported in another major Indian carp, *Cirrhinus cirrhosus*, by Sayeed [54], with a 30.51% higher growth rate in the crossbred groups than in the parental groups. Growth performance in a 2 × 2 diallel cross of wild and cultured giant freshwater prawn, *Macrobrachium rosenbergii*, was assessed by Suburamanian [30]. Weight and length gain of wild and cultured *Macrobrachium rosenbergii* stock shown by Suburamanian [30] have had their antagonistic outcome. However, both length gain and weight gain augmented average heterosis by 0.84% and 6.70%, respectively, in crossbred stock. Hence, diallel cross breeding is a simple way to genetically improve different domesticated stocks and lines in small aquaculture industries.

### 4.3. Myofiber Characteristics of L. ariza

In the early juvenile stage, the trunk skeletal myofiber morphometry of distinct groups of *L. ariza* resulted in an uncounted number of fibers with small-diameter which were further encompassed by large fibers. Hyperplasia-derived new fibers remained small during its’ first generation and the size tended to increase as it grew. As skeletal muscle is the main contributor to body weight in most fish, it is probable that the size of a fish is limited by the growth of this tissue [55]. Muscle histology is an extensively used technique in the research community that resorted to manifest the muscle growth dynamics of *Dicentrarchus labrax* [56], *Dentex dentex* [57], *Gadus morhua* [58], *Sparus aurata* [59], *Solea senegalensis* [60], and many other species (reviewed by Valente [61]).

Hyperplasia, hypertrophy, and elongation are the subsequent events involved in muscle formation and muscle fiber enlargement of teleost fish. Myogenesis consists of serial complex events involving the specification, proliferation, differentiation, migration, and fusion of precursor cells to form multinucleated muscle fibers. The myogenesis rate, the subcellular organelles’ architecture, gene expression patterns, and muscle fibers size and numbers of an organism are all determined by the environment where they belong [62]. The post-hatching growth of the lateral muscle of gilt-head seabream, *Sparus aurata*, was studied by Rowlerson [59], who aimed to identify and quantify muscle fiber hyperplasia and hypertrophy using morphometry. They observed that hyperplastic growth was slow at hatching, but increased during the larval stage by the apposition of new fibers along the proliferation zones. Post-larvally, between 60- and 90-days post-hatching, a new hyperplastic process started in the fast white muscle as nuclei proliferated and new muscle fibers were formed throughout the entire layer. The presence of many small-diameter fibers in the white muscle in our study supports hyperplastic myogenesis during this period (early juvenile stage). Hyperplasia is most obvious when fish grow fastest (i.e., during the larval and early juvenile stages) [63]. In contrast, Rowlerson and Veggetti [41] and Johnston [64] substantiated that muscle hypertrophy is evident in all life stages, with dominance in later stages (juvenile to adult).

In this study, the larval stage of *L. ariza* was the main phage where muscle growth eventuated by way of hyperplasia—ascertained from the appearance of mosaic fiber lines. Among all nine groups, the highest muscle hyperplasia eventuated in G4K♀A♂ (Figure 5) (proven by the presence of the highest percentage of small-diameter fibers) which is a possible consequence of new myotube generation—a sequelae of proliferated satellite cells amalgamation elucidated by Johnston [65] and Dal Pai-Silva [66]. This hyperplasia also leads the increased growth of *L. ariza* of the G4K♀A♂ group, a clear confirmation from the analysis of muscle fiber morphometry.

### 4.4. Microsatellite Variation in Parental and Crossbreed Groups of L. ariza

Genotyping using appropriate molecular markers is used to assess genetic variations in fish populations. In the present study, we created nine groups by applying a 3 × 3 diallel crossing design in three different wild stocks of *L. ariza*. In principle, the genetic variation should be higher in crossbreeds than in pure breeds in terms of heterozygosity, owing to the combination of different alleles. To assess genetic variations in different groups of *L. ariza*, we used eight microsatellite markers (*Lr1*, *Lr2*, *Lr3*, *Lr22*, *Lr24*, *Lr27*, *Lr28*, and *Lr29*), of which two (*Lr24* and *Lr27*) were polymorphic in all groups. Previously, these markers were used in population genetic studies of major Indian carp, where polymorphisms were detected at *Lr3* in *C. cirrhosus* [67] and at *Lr1*, *Lr2*, *Lr3*, *Lr22*, *Lr24*, *Lr28*, and *Lr29* in *L. rohita* [33,68,69]. The use of primers of different species could have caused the deviation of our results from those of previous studies. The allelic richness of G4K♀A♂ in terms of the highest N_a_, indicated that this crossbred group was superior to the other groups. In contrast, the lowest average N_a_ (2.000) was observed in G6A♀K♂, which indicates the allelic deficiency of that group. Overall, low levels of variation in terms of allelic richness and heterozygosity were observed in the three riverine stocks of *L. ariza*, which indicates that the populations might have suffered from a reduction in their sizes, causing bottleneck effects. The loss of alleles and heterozygosity may increase with bottlenecking and inbreeding over generations in hatchery stocks [44]. However, the *L. ariza* samples used in the present study were collected from wild sources. The reduction in allelic variation may also be explained by the bottleneck effect and genetic drift in the population, resulting from a sudden decrease in population size (N_e_), which might have occurred in the case of *L. ariza*.

Populations with less genetic variation have little chance of improvement through selective breeding. In that case, crossbreeding is the most promising alternative that can improve genetic variation and the potential for the genetic improvement of the population. Accordingly, as hypothesized, we observed higher heterozygosities in four (G4K♀A♂, G5K♀J♂, G6A♀K♂, and G7A♀J♂) of the six crossbred groups compared to their parental groups. Therefore, the results of the present study have created an opportunity to improve the genetic quality of existing stocks of *L. ariza* through crossbreeding. Moreover, we observed higher growth in terms of length and weight gain in four of the six crossbreeds compared to that of the original riverine stocks. Zeng [18] also observed higher growth performance and genetic diversity in crossbreed groups than in purebred groups in Chinese perch (*Siniperca chuatsi*), which supports the findings of the present study. In crossbred stocks of *Macrobrachium rosenbergii* from the diallel program, genetic variation increased in an analogy of their cultured stock [30]. Asaduzzaman [1] found higher genetic variation in the Atrai stock of *L. ariza* among three river populations, including Atrai, Jamuna, and Kangsha, one of the parental populations of G4K♀A♂. The Kangsha and Atrai Rivers are geographically distantly located, and there is little chance of genetic interchange between the two stocks. The crossing between the G4K♀A♂ stocks in the present study resulted in genetic intermixing, which could have caused the high genetic variation in G4K♀A♂. The high genetic diversity observed in a population could be explained by the overlap of generations, population mixing from different geographical locations, natural selection favoring heterozygosity, or subdivision accompanied by genetic drift.

The lowest gene flow (0.838) and the highest genetic distance (0.335) were observed in G1K♀K♂ and G3A♀A♂, respectively. These results could be attributed to the geographic isolation of the Atrai and Kangsha Rivers, which minimizes the chances of population exchanges between the two rivers. Thus, the *L. ariza* stock of the Atrai River is genetically distinct from that of the Kangsha River. Along with the stock of Atrai River, populations of Kangsha and Jamuna expressed phenotypic variations [70] and genetic variation owing to their distinct geographic locations. Hasanat [68] found a higher genetic distance between the Halda and Jamuna populations owing to the geographic distance between the two rivers. In contrast, the lowest genetic distance (0.050) was observed between G3A♀A♂ and G6A♀K♂. The main reason for this was the crossbreeding pattern used between the parents of the two groups. It could be suggested that certain genetic effects in addition to heterosis were largely responsible for the relative stock performance in this study; as a consequence, subsequent generations could be improved in terms of selection strategy.

## 5. Conclusions

In this study, we examined for the first time the muscle growth and genetic variation in diallel crosses of *L. ariza* populations, venturing to develop an effective stock improvement method and generate high-quality *L. ariza* seeds. We analyzed 30 individuals from each of the nine groups of *L. ariza* using eight microsatellite primers. This small number of samples is a limitation of the study to elucidate an extensive epilogue on genetic variation of *L. ariza* in Bangladesh. However, based on the results of the present study, it can be inferred that the diallel cross increases the genetic variation in the crossbreeds compared to the purebred *L. ariza.* These results also highlight the natural resources that require scientific management for the preservation of pure breeds (e.g., the Atrai and Kangsha populations based on the performance of G4K♀A♂) in the case of poor broodstock performances of the crossbred populations owing to allelic segregation. This G4K♀A♂ line could be further advanced, improving genetic and phenotypic variation in their subsequent generations following pertinent selection as well as breeding.

## Figures and Tables

**Table 1:** Microsatellite primers used for molecular characterization of Ariza labeo (*L. ariza*).

Locus	Primer Sequence	Annealing Temperature (°C)
*Lr1*	F-GAAAGCTGCTCGTCCTTGAA R-GAAAGCTGCTCGTCCTTGAA	53
*Lr2*	F-GGGTGTGGGAGAGAAAGAGAG R-GGAGTCTGACAAATGCAGCAAG	62
*Lr3*	F-ATCTGGCTGCCTAATCACC R-CATCGGCGACTGCACTGGA	58
*Lr22*	F-GATCTGTGTGTGTGTGTGC R-GGTGGCGACACAACAAATG	58
*Lr24*	F-CAAGGCGAAAAGTGTCCAT R-AAGAAATTGGTAAAGTGTTTC	56
*Lr27*	F-TGGAAATCGAAGGCGTTTCCAC R-AGCACTTACAGTCCATTGGCTC	60
*Lr28*	F-AAAGGAAACAGACTCCATCAG R-CGCTAGCACTTTAATTTCACAGAG	50
*Lr29*	F-CCCACGCAAACTCCTGTT R-GGAACAAGGCCAGAGCTTTA	50

**Table 2:** Growth response and survival rate of three river populations of Ariza labeo (*L. ariza*) during the 12 months domestication period^†^.

Populations	Initial Size		Final Size		Survival Rate (%)
	Length (cm)	Weight (g)	Length (cm)	Weight (g)	
Atrai	5.89 ± 0.85 ^a^	7.08 ± 0.94 ^a^	16.78 ± 0.68 ^a^	95.92 ± 2.24 ^a^	84 ± 0.15 ^a^
Kangsha	5.80 ± 0.73 ^a^	7.06 ± 0.91 ^a^	14.36 ± 1.05 ^b^	90.36 ± 1.88 ^b^	91 ± 0.3 ^b^
Jamuna	5.85 ± 0.92 ^a^	7.01 ± 0.86 ^a^	13.30 ± 3.05 ^b^	89.65 ± 2.46 ^b^	86 ± 0.12 ^a^

^†^ Values are expressed as mean ± SD of three replicates (*n* = 100 per replication). Values within the same row with different or same superscript letters are significantly different (*p* < 0.05) or are not significantly different (*p* > 0.05), respectively (one-way ANOVA).

**Table 3:** Fertilization, hatching and survival rates of three parental and six reciprocal crosses of Ariza labeo (*L. ariza*) during seed production^†^. The single words picked to name the purebred and crossbred groups bear the full forms as G → Group, K → Kangsha River stock, J → Jamuna River stock and A → Atrai River stock.

Parameters	G1K♀K♂	G2J♀J♂	G3A♀A♂	G4K♀A♂	G5K♀J♂	G6A♀K♂	G7A♀J♂	G8J♀K♂	G9J♀A♂
Fertilization rate (%)	92 ± 2.01 ^d^	94 ± 1.09 ^b^	93 ± 1.11 ^c^	95 ± 2.11 ^a^	91 ± 3.03 ^e^	90 ± 1.99 ^f^	92 ± 2.05 ^d^	91 ± 2.21 ^c^	91 ± 1.97 ^e^
Hatching rate (%)	87 ± 1.21 ^b^	84 ± 3.08 ^e^	85 ± 2.08 ^d^	88 ± 1.03 ^a^	86 ± 2.58 ^c^	81 ± 4.01 ^f^	83 ± 1.57 ^f^	82 ± 2.09 ^b^	84 ± 3.01 ^d^
Survival rate (%)	79 ± 1.03 ^d^	76 ± 1.04 ^e^	81 ± 3.44 ^b^	82 ± 1.88 ^a^	80 ± 1.66 ^c^	81 ± 3.77 ^b^	78 ± 2.68 ^b^	80 ± 1.67 ^a^	79 ± 1.22 ^c^

^†^ Values are expressed as mean ± SD of three replicates. Values within the same row with different or same superscript letters are significantly different (*p* < 0.05) or are not significantly different (*p* > 0.05), respectively (one-way ANOVA).

**Table 4:** Growth performance of the fry of three parental and six reciprocal crosses of Ariza labeo (*L. ariza*) after 90 days of rearing in earthen pond condition^†^. The single words picked to name the purebred and crossbred groups bear the full forms as G → Group, K → Kangsha River stock, J → Jamuna River stock and A → Atrai River stock.

Line	Initial Length (cm)	Final Length (cm)	Length Gain (cm)	Initial Weight (g)	Final Weight (g)	Weight Gain (g)	SGR (% Day^−1^)
G1K♀K♂	3.81 ± 0.20	6.30 ± 0.34 ^a^	2.49 ± 0.30 ^a^	0.59 ± 0.20	3.72 ± 0.40 ^a^	3.13 ± 0.20 ^a^	3.07 ± 0.10 ^a^
G2J♀J♂	3.84 ± 0.30	6.28 ± 0.50 ^a^	2.44 ± 0.20 ^a^	0.61 ± 0.30	3.65 ± 0.50 ^a^	3.04 ± 0.20 ^b^	2.98 ± 0.20 ^b^
G3A♀A♂	3.95 ± 0.10	6.35 ± 0.50 ^a^	2.40 ± 0.30 ^a^	0.62 ± 0.20	3.76 ± 0.30 ^a^	3.14 ± 0.30 ^a^	3.00 ± 0.20 ^b^
G4K♀A♂	3.98 ± 0.60	6.65 ± 0.50 ^b^	2.67 ± 0.40 ^b^	0.57 ± 0.20	3.96 ± 0.50 ^b^	3.39 ± 0.20 ^c^	3.23 ± 0.20 ^c^
G5K♀J♂	3.85 ± 0.80	6.42 ± 0.60 ^a^	2.57 ± 0.20 ^c^	0.59 ± 0.20	3.83 ± 0.40 ^c^	3.24 ± 0.10 ^d^	3.12 ± 0.30 ^a^
G6A♀K♂	3.90 ± 0.50	6.53 ± 0.30 ^c^	2.63 ± 0.20 ^b^	0.60 ± 0.10	3.79 ± 0.30 ^c^	3.19 ± 0.30 ^a^	3.07 ± 0.30 ^a^
G7A♀J♂	3.83 ± 0.60	6.30 ± 0.26 ^b^	2.45 ± 0.30 ^a^	0.58 ± 0.30	3.78 ± 0.10 ^a^	3.11 ± 0.20 ^b^	3.05 ± 0.20 ^a^
G8J♀K♂	3.86 ± 0.20	6.36 ± 0.48 ^a^	2.48 ± 0.40 ^b^	0.60 ± 0.40	3.79 ± 0.30 ^b^	3.13 ± 0.30 ^c^	3.15 ± 0.30 ^b^
G9J♀A♂	3.91 ± 0.50	6.41 ± 0.60 ^b^	2.52 ± 0.20 ^c^	0.61 ± 0.50	3.81 ± 0.40 ^c^	3.22 ± 0.40 ^d^	3.08 ± 0.40 ^c^

^†^ Values are expressed as mean ± SD of three replicates. Values within the same row with different or the same superscript letters are significantly different (*p* < 0.05) or are not significantly different (*p* > 0.05), respectively (one-way ANOVA).

**Table 5:** Heterosis for total length (TL), body weight, hatching and survival rate in six cross breed groups of Ariza labeo (*L. ariza*). The single words picked to name the purebred and crossbred groups bear the full forms as G → Group, K → Kangsha River stock, J → Jamuna River stock and A → Atrai River stock.

Trait	Heterosis (%)					
	G4K♀A♂	G5K♀J♂	G6A♀K♂	G7A♀J♂	G8J♀K♂	G9J♀A♂
Length	8.87	6.07	7.19	4.14	5.18	5.61
Weight	24.74	23.10	21.37	20.50	22.29	22.24
Hatching rate	2.30	0.58	−5.81	−1.77	4.00	−0.59
Survival rate	2.50	3.20	1.25	−0.63	3.20	0.63

**Table 6:** Genetic variations at three microsatellite loci (Lr3, Lr24 and Lr27) in nine different groups of Ariza labeo (*L. ariza*). The single words picked to name the purebred and crossbred groups bear the full forms as G → Group, K → Kangsha River stock, J → Jamuna River stock and A → Atrai River stock.

Locus	Parameters	G1K♀K♂	G2J♀J♂	G3A♀A♂	G4K♀A♂	G5K♀J♂	G6A♀K♂	G7A♀J♂	G8J♀K♂	G9J♀A♂
*Lr3*	Na	2.000	1.000	2.000	2.000	2.000	1.000	1.000	2.000	1.000
	Ne	1.800	1.000	1.385	1.385	1.385	1.000	1.000	1.385	1.000
	Ho	0.000	0.000	0.000	0.000	0.000	0.000	0.000	0.000	0.000
	He	0.444	0.000	0.278	0.278	0.278	0.000	0.000	0.278	0.000
	1-(Ho/He)	1.000	N/A	1.000	1.000	1.000	N/A	N/A	1.000	N/A
*Lr24*	Na	2.000	3.000	3.000	4.000	2.000	2.000	3.000	2.000	2.000
	Ne	1.600	2.571	2.323	2.400	1.600	1.832	2.333	1.677	1.786
	Ho	0.167	0.333	0.500	0.833	0.500	0.750	0.540	0.510	0.450
	He	0.375	0.611	0.569	0.583	0.375	0.469	0.420	0.530	0.550
	1-(Ho/He)	0.556	0.455	0.122	−0.429	−0.333	−0.600	−0.321	0.167	0.360
*Lr27*	Na	4.000	5.000	4.000	4.000	4.000	4.000	4.000	4.000	4.000
	Ne	2.057	4.500	3.600	3.130	3.429	2.764	2.650	3.720	3.120
	Ho	0.500	0.667	0.333	0.833	0.833	0.833	0.530	0.610	0.550
	He	0.514	0.778	0.722	0.681	0.708	0.639	0.467	0.649	0.572
	1-(Ho/He)	0.027	0.143	0.538	−0.224	−0.176	−0.304	−0.211	0.143	0.116
Average number of alleles		2.250	2.500	2.500	2.750	2.250	2.000	2.250	2.250	2.500
Average effective number of alleles		1.614	2.268	2.077	1.979	1.853	1.663	1.713	1.816	2.122
Average Ho over loci		0.167	0.250	0.208	0.417	0.333	0.396	0.342	0.249	0.233
Average He over loci		0.333	0.347	0.392	0.385	0.340	0.277	0.316	0.288	0.298
Average 1-(Ho/He) over loci		0.528	0.299	0.553	−0.116	0.163	−0.452	−0.144	0.178	0.211
Mean percentage of polymorphic loci		75%	50%	75%	75%	75%	50%	50%	75%	50%

Na = No. of alleles, Ne = Effective no. of alleles, Ho = Heterozygosity observed, He = Heterozygosity expected, 1-(Ho/He) = Fixation index, N/A = Not applicable.

**Table 7:** Pairwise matrix of Nei’s genetic Distance (Nei, 1972) (below diagonal) and gene flow (Nm) (above diagonal) of nine different groups of Ariza labeo (*L. ariza*). The single words picked to name the purebred and crossbred groups bear the full forms as G → Group, K → Kangsha River stock, J → Jamuna River stock and A → Atrai River stock.

Group	G1K♀K♂	G2J♀J♂	G3A♀A♂	G4K♀A♂	G5K♀J♂	G6A♀K♂	G7A♀J♂	G8J♀K♂	G9J♀A♂
G1K♀K♂	****	1.756	0.838	1.433	1.138	1.247	1.233	1.168	1.323
G2J♀J♂	0.129	****	1.702	1.970	3.943	1.197	1.870	2.978	2.134
G3A♀A♂	0.335	0.217	****	2.323	1.373	7.277	1.886	2.424	1.778
G4K♀A♂	0.265	0.169	0.215	****	1.742	1.411	2.446	1.687	1.534
G5K♀J♂	0.314	0.251	0.053	0.245	****	3.000	3.132	2.863	2.180
G6A♀K♂	0.321	0.212	0.050	0.063	0.237	****	1.664	1.699	3.214
G7A♀J♂	0.295	0.245	0.097	0.155	0.315	0.261	****	2.357	1.876
G8J♀K♂	0.268	0.164	0.123	0.187	0.286	0.225	0.313	****	2.665
G9J♀A♂	0.310	0.198	0.212	0.280	0.317	0.249	0.122	0.220	****

## Data Availability

The data presented in this study are available on request from the corresponding author.

## Supplementary Materials

The following supporting information can be downloaded at: https://www.mdpi.com/article/10.3390/biology11050691/s1, Table S1: Detail information of inbred and crossbred groups.

## References

[1] Asaduzzaman M., Ahammad A.K.S., Haque M.M., Rahman M.A. (2015). Morpho-Genetic Analysis of Three River Populations of Bhagna, *Labeo ariza* (Hamilton 1807) in Bangladesh. J. Aquac. Mar. Biol..

[2] Kottelat M. (2013). The Fishes of the Inland Waters of Southeast Asia: A Catalogue and Core Bibliography of the Fishes Known to Occur in Freshwaters, Mangroves and Estuaries. Supplement No. 27. Raffles Bull. Zool..

[3] Felts R.A., Fajts F., Akteruzzaman M. (1996). Small Indigenous Fish Species Culture in Bangladesh (Technical Brief), IFADEP Sub Project 2, Development of Inland Fisheries.

[4] Rahman A.K.A. (2005). Freshwater Fishes of Bangladesh.

[5] Rahman M.A., Zaher M., Azimuddin K.M. (2009). Development of Fingerling Production Techniques in Nursery Ponds for the Critically Endangered Reba Carp, *Cirrhinus ariza* (Hamilton, 1807). Turkish J. Fish. Aquat. Sci..

[6] IUCN (2015). Red List of Bangladesh: Volume 5: Freshwater Fishes.

[7] Mariappan P., Antony C., Subramaniam B., Nagarahalli M., Bhosale M.M. (2021). Successful Breeding of the Endemic Cyprinid Fish Dawkinsia Rohani in Controlled Condition—First Report. Aquac. Res..

[8] Das P., Behera B.K., Meena D.K., Singh S.K., Mandal S.C., Das S.S., Yadav A.K., Bhattacharjya B.K. (2016). Comparative Efficacy of Different Inducing Agents on Breeding Performance of a near Threatened Cyprinid Osteobrama Belangeri in Captivity. Aquac. Reports.

[9] Borah B.C. (2020). Advancing Sexual Maturation and Induced Breeding of a Minor Carp, *Labeo gonius* (Hamilton, 1822) (Teleostei: Cyprinidae) by Impact of Artificially Enhanced Temperature and Photoperiod in Assam. J. Exp. Zool. India.

[10] Ahammad A.K.S., Gani A., Ahmed M.B.U., Khan M.G.Q., Hossain M.Y., Haque M.M., Ceylan H. (2021). Characterization of Best Growing Line of the Minnow, *Gymnostomus ariza* (Hamilton 1807) through Landmark-Based Morphometric Analysis. Egypt. J. Aquat. Biol. Fish..

[11] Hussain M.G., Mazid M.A., Penman D.J., Gupta M.V., Dey M.M. (2005). Carp Genetic Resources. Carp Genetic Resources for Aquaculture in Asia.

[12] Penman D.J., Penman D.J., Gupta M.V., Dey M.M. (2005). Progress in Carp Genetics Research. Carp Genetic Resources for Aquaculture in Asia.

[13] Kucharczyk D., Nowosad J., Wyszomirska E., Cejko B.I., Arciuch-Rutkowska M., Juchno D., Boroń A. (2020). Comparison of Artificial Spawning Effectiveness of HCG, CPH and GnRHa in Combination with Dopamine Inhibitors in a Wild Strain of *Ide Leuciscus Idus* (L.) in Hatchery Conditions. Anim. Reprod. Sci..

[14] Nowosad J., Kupren K., Biegaj M., Kucharczyk D. (2021). Allometric and Ontogenetic Larval Development of Common Barbel during Rearing under Optimal Conditions. Animal.

[15] Regan T., Bean T.P., Ellis T., Davie A., Carboni S., Migaud H., Houston R.D. (2021). Genetic Improvement Technologies to Support the Sustainable Growth of UK Aquaculture. Rev. Aquac..

[16] Zhou Z., Zhang C., Lu X., Wang L., Hao Z., Li M., Zhang D., Yong H., Zhu H., Weng J. (2018). Dissecting the Genetic Basis Underlying Combining Ability of Plant Height Related Traits in Maize. Front. Plant Sci..

[17] Griffing B. (1956). A Generalised Treatment of the Use of Diallel Crosses in Quantitative Inheritance. Heredity.

[18] Zeng Q., Sun C., Dong J., Tian Y., Ye X. (2017). Comparison of the Crossbreeding Effects of Three Mandarin Fish Populations and Analyses of the Microsatellite Loci Associated with the Growth Traits of F1 Progenies. Int. J. Aquac. Fish. Sci..

[19] Crusio W.E., Kerbusch J.M.L., van Abeelen J.H.F. (1984). The Replicated Diallel Cross: A Generalized Method of Analysis. Behav. Genet..

[20] Gjerde B., Reddy P.V.G.K., Mahapatra K.D., Saha J.N., Jana R.K., Meher P.K., Sahoo M., Lenka S., Govindassamy P., Rye M. (2002). Growth and Survival in Two Complete Diallele Crosses with Five Stocks of Rohu Carp (*Labeo rohita*). Aquaculture.

[21] Bentsen H.B., Gjerde B., Eknath A.E., de Vera M.S.P., Velasco R.R., Danting J.C., Dionisio E.E., Longalong F.M., Reyes R.A., Abella T.A. (2017). Genetic Improvement of Farmed Tilapias: Response to Five Generations of Selection for Increased Body Weight at Harvest in *Oreochromis niloticus* and the Further Impact of the Project. Aquaculture.

[22] Haque M.M., Hasan N.A., Eltholth M.M., Saha P., Mely S.S., Rahman T., Murray F.J. (2021). Assessing the Impacts of In-Feed Probiotic on the Growth Performance and Health Condition of Pangasius (*Pangasianodon hypophthalmus*) in a Farm Trial. Aquac. Rep..

[23] Hasan N.A., Haque M.M., Hinchliffe S.J., Guilder J. (2020). A Sequential Assessment of WSD Risk Factors of Shrimp Farming in Bangladesh: Looking for a Sustainable Farming System. Aquaculture.

[24] Karim M., Keus H.J., Ullah M.H., Kassam L., Phillips M., Beveridge M. (2016). Investing in Carp Seed Quality Improvements in Homestead Aquaculture: Lessons from Bangladesh. Aquaculture.

[25] Łączyńska B., Palińska-Żarska K., Nowosad J., Biłas M., Krejszeff S., Müller T., Kucharczyk D., Żarski D. (2016). Effect of Age, Size and Digestive Tract Development on Weaning Effectiveness in Crucian Carp, *Carassius carassius* (Linnaeus, 1758). J. Appl. Ichthyol..

[26] Ahammad A.K.S., Asaduzzaman M., Asakawa S., Watabe S., Kinoshita S. (2015). Regulation of Gene Expression Mediating Indeterminate Muscle Growth in Teleosts. Mech. Dev..

[27] Johnston I.A., Lee H.T., Macqueen D.J., Paranthaman K., Kawashima C., Anwar A., Kinghorn J.R., Dalmay T. (2009). Embryonic Temperature Affects Muscle Fibre Recruitment in Adult Zebrafish: Genome-Wide Changes in Gene and MicroRNA Expression Associated with the Transition from Hyperplastic to Hypertrophic Growth Phenotypes. J. Exp. Biol..

[28] Asaduzzaman M., Akolkar D.B., Kinoshita S., Watabe S. (2013). The Expression of Multiple Myosin Heavy Chain Genes during Skeletal Muscle Development of Torafugu Takifugu Rubripes Embryos and Larvae. Gene.

[29] Weatherley A.H., Gill H.S. (1989). The Role of Muscle in Determining Growth and Size in Teleost Fish. Experientia.

[30] Suburamanian D., Heo M.S., Chellam B. (2015). Genetic Assessment for Growth Performance in Diallel Cross of Wild and Cultured Giant Freshwater Prawn, *Macrobrachium rosenbergii*. J. Appl. Aquac..

[31] Crooijmans R.P.M.A., Bierbooms V.A.F., Komen J., Van Der Poel J.J., Groenen M.A.M. (1997). Microsatellite Markers in Common Carp (*Cyprinus carpio* L.). Anim. Genet..

[32] DeWoody J.A., Avise J.C. (2000). Microsatellite Variation in Marine, Freshwater and Anadromous Fishes Compared with Other Animals. J. Fish Biol..

[33] Alam M.S., Jahan M., Hossain M.M., Islam M.S. (2009). Population Genetic Structure of Three Major River Populations of Rohu, *Labeo rohita* (Cyprinidae: Cypriniformes) Using Microsatellite DNA Markers. Genes Genom..

[34] Shah M.S., Sutapa S.S., Islam S.S., Rahaman S.M.B., Huq K.A., Rahman M.A. (2017). Heterosis Analysis in Strain-Crossed Hybrid Rohu (*Labeo rohita*) through Microsatellite DNA Variability Assay. Turkish J. Fish. Aquat. Sci..

[35] Dudu A., Georgescu S.E., Costache M., Caliskan M., Oz G.C., Kavaklı İ.H., Ozcan B. (2015). Evaluation of Genetic Diversity in Fish Using Molecular Markers. Molecular Approaches to Genetic Diversity.

[36] Sarder R., Bondad-Reantaso M.G. (2007). Freshwater Fish Seed Resources in Bangladesh. Proceedings of the Assessment of Freshwater Fish Resources for Sustainable Aquaculture.

[37] Ahammad A.K.S., Hasan N.A., Haque M.M., Bashar A., Ahmed M.B.U., Alam M.A., Asaduzzaman M., Bashar M.A., Mahmud Y. (2021). Environmental Factors and Genetic Diversity as Drivers of Early Gonadal Maturation: A Gonadosomatic Index Based Investigation on Indian Shad, *Tenualosa ilisha* Population of Bangladesh. Front. Mar. Sci..

[38] Tave D., Tave D. (1993). Genetics for Fish Hatchery Managers.

[39] Johnston I.A. (1999). Muscle Development and Growth: Potential Implications for Flesh Quality in Fish. Aquaculture.

[40] Humason G.L. (1962). Animal Tissue Techniques.

[41] Rowlerson A., Veggetti A., Johnston I.A. (2001). Cellular Mechanisms of Post-Embryonic Muscle Growth in Aquaculture Species. Fish Physiology: Muscle Development and Growth.

[42] Weatherley A.H., Gill H.S. (1984). Growth Dynamics of White Myotomal Muscle Fibres in the Bluntnose Minnow, Pimephales Notatus Rafinesque, and Comparison with Rainbow Trout, Salmo Gairdneri Richardson. J. Fish Biol..

[43] Patel A., Das P., Barat A., Meher P.K., Jayasankar P. (2010). Utility of Cross-Species Amplification of 34 Rohu Microsatellite Loci in *Labeo bata*, and Their Transferability in Six Other Species of the Cyprinidae Family. Aquac. Res..

[44] Alam M.S., Islam M.S. (2005). Population Genetic Structure of *Catla catla* (Hamilton) Revealed by Microsatellite DNA Markers. Aquaculture.

[45] Peakall R., Smouse P.E. (2012). GenAlEx 6.5: Genetic Analysis in Excel. Population Genetic Software for Teaching and Research—An Update. Bioinformatics.

[46] Roush K.S., Jeffries M.K.S. (2019). Gonadosomatic Index as a Confounding Variable in Fish-Based Screening Assays for the Detection of Anti-Estrogens and Nonaromatizable Androgens. Environ. Toxicol. Chem..

[47] Tucker E.K., Zurliene M.E., Suski C.D., Nowak R.A. (2020). Gonad Development and Reproductive Hormones of Invasive Silver Carp (*Hypophthalmichthys molitrix*) in the Illinois River. Biol. Reprod..

[48] Chapman D.C., Deters J.E. (2009). Effect of Water Hardness and Dissolved-Solid Concentration on Hatching Success and Egg Size in Bighead Carp. Trans. Am. Fish. Soc..

[49] Rahman M.M., Habib M.A., Shah M.S. (2007). Induced Breeding of *Cirrhinus reba* (Ham.) and *Labeo bata* (Ham.). Khulna Univ. Stud..

[50] Lashari P.K., Narejo N.T., Laghari M.Y., Mastoi A.M. (2007). Studies on the Gonadosomatic Index and Fecundity of a Carp *Cirrhinus reba* (Hamilton) from Fishponds of District Jacobabad, Sindh, Pakistan. Pak. J. Zool..

[51] Hossain Q.Z. (2001). Induced Breeding of the Fish Cirrhinus Reba by Pituitary Gland Extract and Survival of Spawn in Nursery Pond. J. Asiat. Soc..

[52] Chattopadhyay N.R., Patra S., Giri S., Naskar A., Roy U. (2013). Low Cost Innovative Technology for Seed Production of *Cirrhinus reba* (Hamilton, 1822) at Backyard of Murshidabad District, West Bengal, by Using Ovaprim. Int. J. Adv. Fish. Aquat. Sci..

[53] Ali F.S., Nazmi H.M., Abdelaty B.S., El-Far A.M., Goda A.M.A.S. (2017). Genetic Improvement of Farmed Nile Tilapia (*Oreochromis niloticus*) through Se-Lective Breeding in Egypt. Int. J. Fish. Aquat. Stud..

[54] Sayeed M.A. (2015). Bin Strain Crossing in Mrigal (*Cirrhinus cirrhosus*): An Avenue to Persuade Heterosis in F1 Generation of Wild×hatchery Hybrid. J. Fish..

[55] Zimmerman A.M., Lowery M.S. (1999). Hyperplastic Development and Hypertrophic Growth of Muscle Fibers in the White Seabass (*Atractoscion nobilis*)—Zimmerman—1999—Journal of Experimental Zoology—Wiley Online Library. J. Exp. Zool..

[56] Alami-Durante H., Olive N., Rouel M. (2007). Early Thermal History Significantly Affects the Seasonal Hyperplastic Process Occurring in the Myotomal White Muscle of Dicentrarchus Labrax Juveniles. Cell Tissue Res..

[57] Albors O.L., Arizcun M., Abellán E., Blanco A., Ayala M.D., Pastor L.M., Latorre R. (2010). Posthatch Development of the Axial Musculature of the Common Dentex *Dentex dentex*, L (Teleostei). Histol. Histopathol..

[58] Johnston I.A., Andersen Ø. (2008). Number of Muscle Fibres in Adult Atlantic Cod Varies with Temperature during Embryonic Development and Pantophysin (PanI) Genotype. Aquat. Biol..

[59] Rowlerson A., Mascarello F., Radaelli G., Veggetti A. (1995). Differentiation and Growth of Muscle in the Fish *Sparus aurata* (L): II. Hyperplastic and Hypertrophic Growth of Lateral Muscle from Hatching to Adult. J. Muscle Res. Cell Motil..

[60] Campos C., Valente L.M.P., Conceição L.E.C., Engrola S., Sousa V., Rocha E., Fernandes J.M.O. (2013). Incubation Temperature Induces Changes in Muscle Cellularity and Gene Expression in Senegalese Sole (*Solea senegalensis*). Gene.

[61] Valente L.M.P., Moutou K.A., Conceição L.E.C., Engrola S., Fernandes J.M.O., Johnston I.A. (2013). What Determines Growth Potential and Juvenile Quality of Farmed Fish Species?. Rev. Aquac..

[62] Johnston I.A. (2006). Environment and Plasticity of Myogenesis in Teleost Fish. J. Exp. Biol..

[63] Higgins P.J., Thorpe J.E. (1990). Hyperplasia and Hypertrophy in the Growth of Skeletal Muscle in Juvenile Atlantic Salmon, *Salmo salar* L.. J. Fish Biol..

[64] Johnston I.A., Bower N.I., Macqueen D.J. (2011). Growth and the Regulation of Myotomal Muscle Mass in Teleost Fish. J. Exp. Biol..

[65] Johnston I.A., Alderson R., Sandham C., Dingwall A., Mitchell D., Selkirk C., Nickell D., Baker R., Robertson B., Whyte D. (2000). Muscle Fibre Density in Relation to the Colour and Texture of Smoked Atlantic Salmon (*Salmo salar* L.). Aquaculture.

[66] Dal Pai-Silva M., Carvalho R.F., Pellizzon C.H., Dal Pai V. (2003). Muscle Growth in Nile Tilapia (*Oreochromis niloticus*): Histochemical, Ultrastructural and Morphometric Study. Tissue Cell.

[67] Hasanat M.A., Mollah M.F.A., Alam M.S. (2015). Microsatellite DNA Marker Analysis Revealed Low Levels of Genetic Variability in the Wild and Captive Populations of *Cirrhinus cirrhosus* (Hamilton) (Cyprinidae: Cypriniformes). Biotechnol. J. Int..

[68] Qadeer I., Abbas K. (2017). Microsatellite Markers Based Genetic Structure of Rohu (*Labeo rohita*) in Selected Riverine Populations of Punjab, Pakistan. Pakistan J. Agric. Res..

[69] Tonny U.S., Faroque A.A., Sarder R.I., Mollah F.A. (2014). Assessment of Genetic Variation of Wild Rohu *Labeo rohita* (Hamilton 1822) Populations Using Microsatellite Markers. African J. Fish. Sci..

[70] Ahammad A.K.S., Ahmed M.B.U., Akhter S., Hossain M.K. (2018). Landmark-Based Morphometric and Meristic Analysis in Response to Characterize the Wild Bhagna, *Labeo ariza* Populations for Its Conservation. J. Bangladesh Agric. Univ..

