# Supplementary material for: Diallel Cross Application and Histomolecular Characterization: An Attempt to Develop Reference Stock of Labeo ariza
Source: Biology (Basel). 2022 Apr 30;11(5):691. doi: 10.3390/biology11050691 (PMC9138064; doi:10.3390/biology11050691)
Supplement: Supplementary file 1 [file biology-11-00691-s001.zip › Supplementary file.pdf]

Table S1. Detail information of inbred and crossbred groups.

Group name	Description
G1K♀K♂	G stands for Group and K♀K♂ reflect that the cross between male and female fish from Kangsha stock
G2J♀J♂	G stands for Group and J♀J♂ reflect that the cross between male and female fish from Jamuna stock
G3A♀A♂	G stands for Group and A♀A♂ reflect that the cross between male and female fish from Atrai stock
G4K♀A♂	G stands for Group and K♀A♂ reflect that the cross between male and female fish from Kangsha and Atrai stock, respectively
G5K♀J♂	G stands for Group and K♀J♂ reflect that the cross between male and female fish from Kangsha and Jamuna stock, respectively
G6A♀K♂	G stands for Group and A♀K♂ reflect that the cross between male and female fish from Atrai and Kangsha stock, respectively
G7A♀J♂	G stands for Group and A♀J♂ reflect that the cross between male and female fish from Atrai and Jamuna stock, respectively
G8J♀K♂	G stands for Group and J♀K♂ reflect that the cross between male and female fish from Jamuna and Kangsha stock, respectively
G9J♀A♂	G stands for Group and J♀A♂ reflect that the cross between male and female fish from Jamuna and Kangsha stock, respectively
